# Online EEG-Based Workload Adaptation of an Arithmetic Learning Environment

**DOI:** 10.3389/fnhum.2017.00286

**Published:** 2017-05-30

**Authors:** Carina Walter, Wolfgang Rosenstiel, Martin Bogdan, Peter Gerjets, Martin Spüler

**Affiliations:** ^1^Department of Computer Engineering, Eberhard-Karls University TübingenTübingen, Germany; ^2^Department of Computer Engineering, University of LeipzigLeipzig, Germany; ^3^Knowledge Media Research CenterTübingen, Germany

**Keywords:** Passive brain-computer interface (BCI), Cognitive workload, Electroencephalography (EEG), Online Adaptation, Neurotutor, tutoring system, closed-loop workload adaptation

## Abstract

In this paper, we demonstrate a closed-loop EEG-based learning environment, that adapts instructional learning material online, to improve learning success in students during arithmetic learning. The amount of cognitive workload during learning is crucial for successful learning and should be held in the optimal range for each learner. Based on EEG data from 10 subjects, we created a prediction model that estimates the learner's workload to obtain an unobtrusive workload measure. Furthermore, we developed an interactive learning environment that uses the prediction model to estimate the learner's workload online based on the EEG data and adapt the difficulty of the learning material to keep the learner's workload in an optimal range. The EEG-based learning environment was used by 13 subjects to learn arithmetic addition in the octal number system, leading to a significant learning effect. The results suggest that it is feasible to use EEG as an unobtrusive measure of cognitive workload to adapt the learning content. Further it demonstrates that a promptly workload prediction is possible using a generalized prediction model without the need for a user-specific calibration.

## 1. Introduction

Currently, there is an ongoing debate in mathematics education research on how to optimally support learners' during arithmetic learning (e.g., Askew, 2015; Calder, 2015). Obviously, learning outcome is most promising if the training program and learning content is tailored to the learner's specific needs (e.g., Gerjets and Hesse, 2004; Richards et al., 2007; for numerical interventions see Dowker, 2004; Karagiannakis and Cooreman, 2014). To optimally support the learner's efforts, learning content should neither be too easy, nor too difficult. Therefore, it is crucial for successful learning to keep the cognitive workload in the individual optimal range for each learner (Sweller et al., 1998; Gerjets et al., 2009). This can be achieved by adapting the difficulty of the learning content to the individual competencies of the learner.

Computer-supported learning (Kirschner and Gerjets, 2006) seems specifically suited for implementing adaptivity, because it is easy to implement algorithms that change the difficulty of the presented material based on the learner's behavioral response. This allows for an easy personalization of the learning environment to the user's individual needs, which is assumed to be necessary for efficient learning. So far, adaptive computer-supported learning environments rely on the user's interaction behavior for adaptation, e.g., error-adaptive systems, which change the task difficulty based on the number of erroneous responses (Corbett, 2001; Graesser and McNamara, 2010; Käser et al., 2013). However, such behavioral measures are rather indirect and not very specific with respect to the cognitive processes required for performing the task at hand. For instance, more errors in a row may not only be caused by the difficulty of the task itself but also by task-unspecific processes (e.g., lapses of attention, fatigue, or disengagement).

It was recently proposed to measure cognitive processes directly to enhance human-computer interaction (Zander and Kothe, 2011). This approach, called passive brain-computer interface, could also be applied to improve arithmetic learning environments. Measuring neural correlates of specific cognitive processes allows for a more direct and implicit monitoring of the learner's cognitive state and should thereby allow for a better adaptation of the training content to improve the learning success of the user (Gerjets et al., 2014).

One cognitive state that is important within this context, is the working memory load, or in short workload. Throughout this paper, the term workload will be used as the amount of mental resources that are used to execute a specific task (based on Gevins and Smith, 2006). Working memory describes the small amount of information that can be stored and manipulated in mind simultaneously for the execution of a current cognitive task (Cowan, 2014). As the capacity for storing information at a time is limited, workload describes the extent to which this capacity is used or to which extent the working memory is filled. According to the cognitive load theory (Sweller et al., 1998), this is a bottleneck in learning, as the learning process is hindered if the amount of information the learner has to process exceeds the capacity of the working memory storage. Consequently, if the working memory load can be measured, this allows to adapt the presented learning content in a way that the storage capacities are never exceeded and the workload is always in an optimal range.

The amount of cognitive workload can be measured by Electroencephalography (EEG), which has been shown by multiple studies (Gevins et al., 1997; Murata, 2005; Berka et al., 2007; Wang et al., 2012). Basically, the amount of workload is reflected in two components of the EEG: the power spectrum and event-related potentials. Regarding the effect of workload on event-related potentials, two types of stimuli can be distinguished, task-independent as well as task-dependent stimuli. Using the n-back tasks for task-dependent stimuli, an increase in workload leads to a diminished P300 amplitude (Scharinger et al., 2017). Also for more complex tasks which include task-dependent as well as task-independent stimuli, like a piloting task, it was shown that the P300 amplitude is lower in high workload conditions (Causse et al., 2015). Brouwer et al. (2012) stated this diminishing effect in the P300 amplitude for task-independent stimuli. Besides the P300, also other components of the event-related potential were shown to be sensitive to workload, like the N100 (Ullsperger et al., 2001) or the N200 and mismatch negativity (Kramer et al., 1995), using task-independent stimuli. In Roy et al. (2016a) a significant decrease of the P200 component was stated when workload increased. While ignoring infrequent task-independent auditory probes, they were able to assess mental workload efficiently.

Further, the oscillatory activity in EEG is also affected by workload. Pesonen et al. (2007) have shown that there are workload related changes in theta-, alpha-, and beta-band. Brouwer et al. (2012) also found an increase in frontal theta power and a decrease in occipital alpha power in an n-back task. Specific to arithmetic tasks, it was shown that the cognitive demand results in an increasing power of the theta band and a decreasing power in the alpha band (Harmony et al., 1999).

Regarding an EEG-based prediction of workload, Kohlmorgen et al. (2007) have presented a real-time system in which the workload induced by mental calculation task while driving could be predicted. Brouwer et al. (2012) classified high- against low workload in an n-back task and achieved classification accuracies above 80% using either spectral features, ERPs or a combination of both. Roy et al. (2016b) also compared the classification accuracy of power spectrum and ERP features during a Sternberg memory task and achieved a low performance (60%) with spectral data, while achieving high performance (91%) with ERP data. In a previous study (Walter et al., 2014), we tried to predict the difficulty of arithmetic tasks based on theta, alpha and beta power and achieved an average correlation coefficient of up to 0.88. In a following study (Spüler et al., 2016), we improved the cross-subject prediction and trained a prediction model that works across subjects yielding an average correlation coefficient of 0.82.

While there is enough evidence showing that an EEG-based workload prediction is possible and can be implemented in a real-time system, it has not been used for a closed-loop adaptation in an online learning environment, to the best of our knowledge. In this paper, we show how we developed a learning environment for arithmetic exercises that adapts the task difficulty based on learner's cognitive workload as predicted from the EEG. To concentrate on the usability of the EEG-based learning environment, we wanted a system that works out-of-the-box without the need for a subject specific calibration phase. As we also wanted a stimulus-independent system, only the power spectrum was used for prediction. In the following, we present the EEG-based workload prediction and describe its application in the EEG-based learning environment. To show the feasibility of the EEG-based learning environment, the learning effect for 13 subjects testing this environment is compared to a control group using an error-based learning environment.

## 2. Methods

For the development of an EEG-based workload environment, the work was split in two studies. In the first study, EEG data were collected while subjects solved arithmetic tasks of varying difficulty. Based on this data a prediction model was created that predicts the amount of workload based on the user's EEG. In the second study, which is the main part of this paper, the prediction model was used to estimate a learner's workload online and adapt the task difficulty accordingly. To evaluate the EEG-based learning environment, subjects used it to learn arithmetic addition in the octal number system (e.g., 3 + 5 = 10) and the learning success was compared to a control group, who learned the same task using a learning environment that adapts based on the number of correct responses.

### 2.1. Task design

The participants solved addition tasks with diverse levels of difficulty, which where presented on a desktop computer with a 19 inch TFT display. In the first study, addition tasks in the decimal number system were presented, while the participants had to learn addition in the octal number system in the second study. The difficulty level of the presented addition exercises was defined by their *Q*-value (Thomas, 1963), which reflects the information content of an arithmetic task. This difficulty measurement takes into account both, problem size and the need for a carry over operation, which are the main parameters for problem difficulty in addition.

A more detailed description on calculating the *Q*-value can be found in Thomas (1963) or in Spüler et al. (2016), where also some examples are shown. The addition problems presented in this work were ranging from *Q* = 0.6 (easy, single-digit, e.g., 1 + 1) to *Q* = 7.2 (difficult, four-digit, e.g., 3721 + 1452).

**Figure 1:**
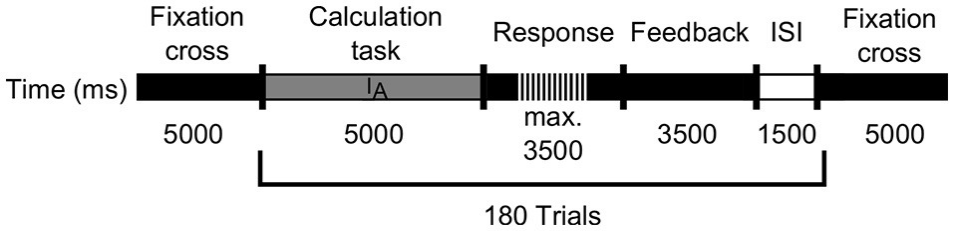
Schematic flow of the learning phase for the EEG-based, as well as the error-based adaptive learning environment. The gray line indicates the calculation phase (*I*_*A*_), whereas the black dashed line represents the response interval. Subsequently a feedback phase occurs indicated by the black line, followed by an inter-stimulus interval (*ISI*) shown as white line.

Each trial, in which one arithmetic problem should be solved, consisted of four phases, which are depicted in Figure 1. First, the calculation phase occurred, where the problem to be solved was shown for 5 s. Subsequently, subjects had a maximum of 3.5 s to type in their result. In the first study, with arithmetic's in the decimal number system, the subjects did not receive feedback. In the second study, the subjects had to learn arithmetic's in the octal number system and therefore got feedback by presenting the correct answer for 3.5 s. Each trial ended with an inter-trial interval (ISI) of 1.5 s, resulting in a total length of approximately 45 min. To avoid the prediction model of being based on perceptual-motor confounds, the time windows used for analyzing EEG data should not contain motor events. As typing in the answer leads to motor artifacts, the calculation phase was used for EEG analysis only.

### 2.2. EEG recording

**Figure 2:**
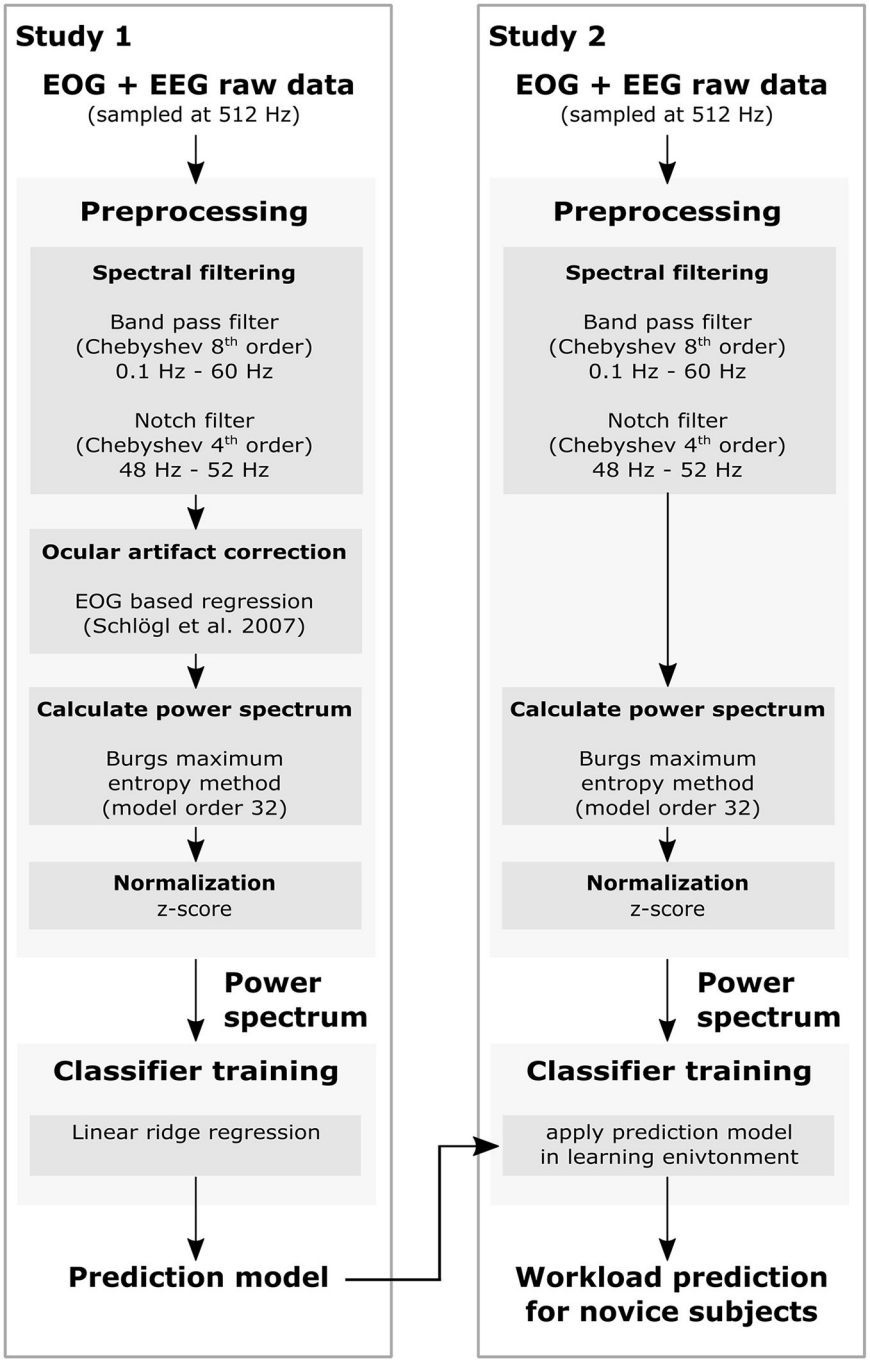
Flow chart of the EEG signal processing pipeline to generate a classifier for workload prediction, used in both studies.

A set of 28 active electrodes (actiCap, BrainProducts GmbH), was used to record EEG signals. They were attached to the scalp, placed according to the extended international electrode 10 − 20 placement system (FPz, AFz, F3, Fz, F4, F8, FT7, FC3, FCz, FC4, FT8, T7, C3, Cz, C4, T8, CPz, P7, P3, Pz, P4, P8, PO7, POz, PO8, O1, Oz, and O2). Three additional electrodes were used to record an electrooculogram (EOG); two of them were placed horizontally at the outer canthus of the left and right eye to measure horizontal eye movements and one was placed in the middle of the forehead between the eyes to measure vertical eye movements. Ground and reference electrodes were placed on the left and right mastoids. EOG- and EEG-signals were amplified by two 16-channel biosignal amplifier systems (g.USBamp, g.tec) and sampled at a rate of 512 Hz and the impedance of each electrode was less than 5*kΩ*. EEG data were band-pass filtered between 0.5 and 60 Hz with a Chebyshev filter of order 8 during the recording. Furthermore, a notch-filter (Chebyshev, order 4) was applied between 48 and 52 Hz to filter out power line noise. The signal processing pipeline is documented in detail in Figure 2.

### 2.3. Study 1: workload during decimal arithmetic tasks

#### 2.3.1. Study design and participants

Ten students (4 male and 6 female; range: 17 − 32 years, *M* = 24.9 years, *SD* = 5.3 years) participated voluntarily in this study and received monetary compensation for participation. The study was approved by the local ethics committee of the Medical Faculty at the University of Tübingen and written informed consent was obtained by the participants. All participants reported normal or corrected to normal vision and no mathematical problems. Participants were chosen randomly. Nevertheless, all were university students (with different fields of study) and can thus be considered as having a high educational background.

Each participant had to solve 240 addition exercises in the decimal number system while EEG was measured. The exercises were presented with an increasing difficulty due to learning effects. If a subject learns and gets better at performing the tasks, exercises with the same objective difficulty (*Q*) can lead to a different perceived difficulty at the start (before learning) and at the end of the session (after learning). If exercises are presented in increasing difficulty, the relationship that a task with higher perceived difficulty has also a higher objective difficulty (*Q*) still holds.

However, it should be noted that the increasing task difficulty over times leads to potential confounds, as there might also be changes in EEG over time that are not related to task difficulty. The only possibility to counter these confounds would be a randomization of the task difficulty, which in turn would corrupt the labels, as the relationship between Q and the perceived difficulty would change over time due to learning effects. Since we needed reliable labels for the training of the prediction model, we decided against a randomization.

#### 2.3.2. Analysis of EEG data

EEG data were corrected for eye-movements using an EOG-based regression method (Schlögl et al., 2007). Furthermore, Burg's maximum entropy method (Cover and Thomas, 2006) with a model order of 32 was used to estimate the power spectrum from 1 to 40 Hz in 1 Hz bins. After calculating the power spectrum, the data was *z-score* normalized along the channels to correct for inter-subject variability in the subjects baseline EEG power. To analyze which electrodes and frequencies change with increasing task difficulty, the signed squared correlation coefficients (sign (*R*) ^*^ *R*^2^) between the power at each frequency bin (for each electrode), as well as *Q*-values of the corresponding trials were calculated.

### 2.4. Study 2: EEG-based learning environment for octal arithmetic tasks

#### 2.4.1. Study design and participants

The participants in the second study were divided into two groups: an experimental group using the EEG-based learning environment and a control group using an error-adaptive learning environment. In both groups, the subjects learned arithmetic addition in the octal number system (e.g., 5 + 3 = 10), which was a completely new task to all subjects.

To evaluate the learning success, each subject did a pre-test and a post-test, before and after using the learning environment for approximately 45 min (180 exercises). The tests consisted of 11 exercises, with varying difficulty. Although difficulty of the exercises were the same for pre- and post-test, the exercises itself were different.

The participants of both groups were university students of various discipline, reported to have normal or corrected to normal vision and participated voluntarily in the EEG experiment. 13 subjects (7 male and 6 female; range 21 − 35 years, *M* = 28.1 years, *SD* = 4.3 years) participated in the experimental group using the EEG-based learning environment. The control group consisted of 11 subjects (7 male and 4 female; range 22 − 27 years, *M* = 23.4 years, *SD* = 1.4 years), using an error-adaptive learning environment which is state of the art. The study was approved by the local ethics committee of the Medical Faculty at the University of Tübingen and written informed consent was obtained by the participants.

#### 2.4.2. Cross-subject regression for online workload prediction

Based on the EEG data from study 1, we created a prediction model that was used to predict the cognitive workload in a timely manner and further was able to adjust the learning environment accordingly. In terms of usability, we wanted the EEG-based learning environment to be useable out-of-the-box without the need for a subject-specific calibration phase, which is why a cross-subject regression method as presented by Walter et al. (2014) was applied.

Therefore, EEG data obtained in study 1 were used for training a linear ridge regression model with a regularization parameter of λ = 10^3^. For training the ridge regression, we used the MATLAB function *ridge* and the regularization parameter was found to be optimal in the previous study, where it was determined by cross-validation. The number of electrodes used for online adaptation was reduced to 16 inner electrodes (FPz, AFz, F3, Fz, FC3, FCz, FC4, C3, Cz, C4, CPz, P3, Pz, P4, Oz, and POz), to be consistent with the electrode positions used in the previous cross-subject study (Walter et al., 2014), where the outer electrodes were not used as those are more prone to artifacts and contain less relevant information. Furthermore, only trials with a *Q*-value smaller than 6 were used to train the regression model, since trials with higher *Q*-value showed similar EEG patterns as very easy trials, which is most likely due to a disengagement of the subjects (Spüler et al., 2016). The power spectrum was calculated for the 5 s time frame of the calculation phase using the Burg's maximum entropy with a model order of 32. To correct for inter-subject variability in the subjects baseline EEG power, the data was *z-score* normalized along the channels. For the final prediction output, we calculated the moving average with a window length of 6 trials in 1 trial steps, which still guaranteed a response time smaller than 1 min of the system. This delay is feasible for the detection of workload since it is not recommendable to adapt an online learning environment too fast (i.e., single trial duration). The moving average also leads to a more robust prediction (Walter et al., 2014), but makes the system react slower to sudden changes in workload, which is feasible since it is not recommendable to adapt the difficulty of an online learning environment too rapidly.

In contrast to study 1, we did not use an EOG-correction in this study. Although it is technically possible, it would not fit with our approach to build an out-of-the-box system, as training data for the EOG-correction would be needed for each subject. This would cost additional time before the user can use the system (and start learning) and therefore would decrease usability of the system.

The so trained regression model was then applied in the EEG-based learning environment, to predict the amount of cognitive workload for novice subjects online.

#### 2.4.3. Online adaptation of the EEG-based learning environment

For the experimental group, the EEG data served as workload indicator. Therefore, we used the output of the previously trained regression model to predict the current workload state of each learner and differentiated three difficulty levels. If the predicted workload was less than *Q* = 0.8, the presented task difficulty was assumed to be too easy. Thus the following *Q*-value was increased by 0.2. Vice versa, the target *Q* of the subsequent task decreased by 0.2 when the predicted workload was greater than *Q* = 3.5. In this case, the presented task difficulty was assumed to be too difficult. If the predicted workload was between *Q* = 0.8 and *Q* = 3.5, the *Q*-value for the next presented task remained the same and the difficulty level was kept constant. These thresholds were defined based on the results in Spüler et al. (2016). Trials with *Q* < 1 were solved correctly in all cases, while none of the subjects were able to solve trials with a *Q* > 6. 50% of the trials with a *Q* = 3.5 were successfully solved on average. The learning session for the experimental group started with an exercise of difficulty level *Q* = 2.

#### 2.4.4. Adaptation of the error-based learning environment

For the control group, an error-adaptive learning environment was used. The number of wrong answers served as performance and adaptation measure. When subjects solved five consecutive tasks correctly, the difficulty level (*Q*) increased by 1. Vice versa, the difficulty level decreased by 1 when participants made three errors in a row. Otherwise, the *Q*-value did not change and the difficulty level was held constant. The adaptation scheme was kept similar in the control group, as in common tutoring systems. The learning session for the control group started with an exercise of difficulty level *Q* = 2.

#### 2.4.5. Evaluating learning success

To compare the learning success of the two groups, the learning effect after completing the learning phase serves as performance measure and is used as an indicator of how successful each subject was supported during learning. Hence, each subject had to perform a pre-test before the learning phase started. This was used to assess the prior knowledge of each user. After the learning session, each participant had to solve a post-test, and the difference in score between the two tests served as indicator of the learning effect. Results of the pre- and post-tests were compared statistically using a two-sided Wilcoxon's ranksum test.

## 3. Results

### 3.1. Study 1: workload related effects in EEG data

As results from study 1 were already published in Spüler et al. (2016), only the most important results relevant for the implementation of the online learning environment are shown here. For a more detailed analysis, we refer to the original publication.

**Figure 3:**
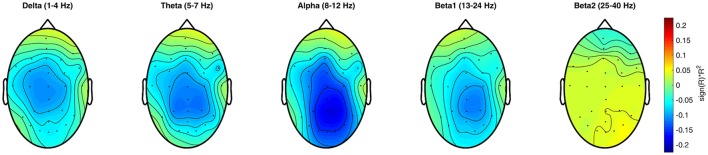
Topographic plots of the signed squared correlation coefficient averaged over all subjects between the frequencies in each band for each electrode and the task difficulty as indicated by the *Q*-value.

Results from the analysis of the EEG data regarding the association between the *Q*-value and the power at each electrode and frequency bin, the *R*^2^-values are shown in Figure 3. In the delta frequency band, there is a small difficulty-related effect over the central electrodes, while the effect in the theta, alpha, and beta frequency band is located over the parieto-occipital electrodes. This effect was strongest for the alpha band (8 − 12*Hz*). While the lower beta band (13 − 24*Hz*) still shows some effects related to task difficulty, they cannot be observed in the upper beta band (25 − 40*Hz*).

### 3.2. Study 2: task performance results

The behavioral results, how well the subjects performed the octal arithmetic task, are shown in Table 1 for both groups. For the experimental group, 45.5% of the 180 exercises were solved correctly on average. Averaged over all subjects, a maximum *Q*-value of 5.85 was reached by using the EEG-based learning environment. Each subject achieved at least the difficulty level of *Q* = 3.2.

**Table 1:** Task performance for all subjects.

**EEG-based learning**	**S01**	**S02**	**S03**	**S04**	**S05**	**S06**	**S07**	**S08**	**S09**	**S10**	**S11**	**S12**	**S13**	**mean**
Correct	38.3	88.9	60.6	10.0	28.9	25.6	31.1	17.8	66.1	33.3	16.7	92.2	82.2	45.5
max. *Q*	6.6	3.2	6.6	6.6	6.6	6.6	6.6	6.6	5.2	6.6	6.6	3.6	4.6	5.85
**Control group**	**S14**	**S15**	**S16**	**S17**	**S18**	**S19**	**S20**	**S21**	**S22**	**S23**	**S24**			**mean**
Correct	64.4	62.2	57.2	66.7	63.3	61.1	63.3	67.8	65.0	66.1	66.7			64.0
max. *Q*	6	4	5	6	4	5	4	4	4	5	4			4.64

*The relative amount of correctly solved trials in % and the maximum Q-value for each subject, as well as the group average are shown. Results of the experimental group using the EEG-based learning environment are displayed at the top, while results from the control group using an error-adaptive learning environment are displayed at the bottom*.

The control group answered 64% of all 180 assignments correctly on average. Since the error-rate was used for adapting the difficulty level of the presented learning material, the number of correctly solved trials was similar across subjects. On average, a maximum *Q*-value of 4.64 was reached (see Table 1). The best subjects reached a maximum *Q*-value of 6, whereas each participant achieved at least the difficulty level of *Q* = 4.

### 3.3. Study 2: learning effect using adaptive learning environments

To evaluate if the EEG-based learning environment works and how it compares to an error-adaptive learning environment, we analyzed the learning effect of each subject by pre- and post-tests. Furthermore, the learning effects between the experimental and the control group were compared.

Table 2 reports the learning effect of each individual subject, as well as the group averages for both groups. After using the EEG-based learning environment and thus learning how to calculate in an octal number system, a learning effect can be recognized for almost every subject of the experimental group, except for subject S02 and S05. On average, 5.08 assignments from 11 post-test tasks were solved correctly after completing the learning phase, 3.54 more assignments compared to the pre-test. On average, a significant learning effect can be verified between the pre- and post-test (*p* = 0.0026, two-sided Wilcoxon test).

**Table 2:** Number of correctly solved trials in the pre-and post-test, as well as the difference, which indicates the learning success for the individual subjects and the group means.

**EEG-based learning**	**S01**	**S02**	**S03**	**S04**	**S05**	**S06**	**S07**	**S08**	**S09**	**S10**	**S11**	**S12**	**S13**	**mean**
Pre-Test	3	0	6	0	2	1	0	1	0	5	1	1	0	1.54
Post-Test	7	0	9	8	2	3	3	2	8	7	7	2	8	5.08
Difference	4	0	3	8	0	2	3	1	8	2	6	1	8	3.54
**Control group**	**S14**	**S15**	**S16**	**S17**	**S18**	**S19**	**S20**	**S21**	**S22**	**S23**	**S24**			**mean**
Pre-Test	4	3	1	0	2	1	2	1	1	2	0			1.55
Post-Test	8	2	4	7	5	8	3	4	4	6	4			5
Difference	4	−1	3	7	3	7	1	3	3	4	4			3.45

*Results of the experimental group using the EEG-based learning environment are displayed at the top, while results from the control group using an error-adaptive learning environment are displayed at the bottom*.

The control group solved 1.55 tasks from the 11 pre-test assignments on average correctly. As the difference to the experimental group is not significant (*p* > 0.05, two-sided Wilcoxon test), equal prior knowledge can be implied for both groups. The best subject of the control group performed 36.36 % of the pre-test tasks accurately, whereas the worst subjects gave no correct answers. For almost every subject, a learning effect for calculating in the octal number system is noticeable after the error-adaptive learning session, except for subject S15. On average, 3.45 more tasks were solved correctly in the post-test, compared to the pre-test, which also shows a significant learning effect between the pre- and post-test (*p* = 0.0016, two-sided Wilcoxon test).

Although the learning effect for the experimental group using the EEG-based learning environment is higher than for the control group, this difference is not significant (*p* > 0.05, two-sided Wilcoxon test).

## 4. Discussion

In the presented work, we have shown that EEG can be used to measure a learner's workload online and adapt a learning environment accordingly. By using a cross-subject regression method, a subject-specific calibration phase can be omitted. Although the cross-subject prediction model was build using data recorded while subjects did arithmetics in the decimal number system, it could be successfully applied to predict the workload during learning of arithmetics in the octal number system.

In the following, the benefits and drawbacks, as well as further ideas for adaptive learning environments will be discussed.

### 4.1. Evaluating an EEG-based learning environment

Commonly, if neural signals (like EEG) are used to estimate a user's mental state or a user's intention, the performance of the prediction method is assessed in terms of accuracy, correlation or other metrics (Spüler et al., 2015) that try to quantify how well the prediction model is working. While we have also evaluated the prediction performance of our model in a previous publication (Spüler et al., 2016), this kind of assessment is no more feasible in the here presented scenario with an online learning environment.

The reason for this is the lack of an objective measure for the user's workload. For the creation of the prediction model we used EEG data from a task that all subjects were able to do fluently (addition in decimal system). As no learning effects are expected in this case, the difficulty of the task (measured by Q) was used as a subjective measure of expected workload. For the online learning environment, the relationship between task difficulty and workload does not hold anymore, as the learning environment induces learning effects. At the beginning, when the task is unknown to the user, even easy exercises will induce a high workload. After using the learning environment, the user may have mastered arithmetic tasks in the octal number system, and even exercises with moderate difficulty will result in a low workload. As the relationship between task difficulty and workload changes in the course of learning, the predicted task difficulty cannot be used for performance evaluation in an online learning environment.

As the task difficulty measured by the *Q*-value is the only objective measure we have, and the relationship to workload is invalidated in the online scenario, we have no means to objectively assess the prediction performance of our model. If the reader is interested in an assessment of the model performance, we refer to our previous publication (Spüler et al., 2016), where the model was evaluated on offline data.

Although we cannot assess the performance of the prediction model in the online scenario, we can evaluate the EEG-based learning environment with regards to its effect on the learning success. Learning success was defined as the difference in score between the pre- and post-tests, which were done by the subjects before and after using the learning environment and is a common measure for the evaluation of learning environments (Chi et al., 2011). As it is not important for the learner how accurate the workload-prediction works, but it is important how much the learning success can be improved, learning success is also the most user-centered metric.

Due to these facts, that other commonly used metrics are not applicable for the scenario of an online learning environment and that learning success is the most user-centered metric, we used the learning success as prime outcome measure for this study.

### 4.2. Proving the concept of an EEG-based learning environment

As subjects using the EEG-based learning environment showed a significant learning effect, this work is a successful proof-of-concept that an EEG-based learning environment works. So far, the use of EEG in a reading tutor has been investigated (Chang et al., 2013) or the cognitive and emotional state of the user is modeled to improve a tutoring system (Graesser et al., 2007), but to the best of our knowledge, this work presents for the first time a closed-loop system using an EEG-based workload adaptation in an arithmetic learning environment.

When comparing the experimental group using the EEG-based learning environment with the control group using the error-based learning environment, the learning success was higher for the experimental group, but the difference was not significant. As this study only compared an EEG-adaptive system to an error-adaptive system, it would be interesting for future studies to compare an EEG-based system against non-adaptive learning environments to see how it compares to those and if a significant performance difference can be achieved. Nevertheless, the results achieved with this study indicate that an EEG-based learning environment is an alternative to the state-of-the-art approach, but usability of the system is still an open issue.

Although, we aimed at a high usability for the presented system by using a cross-subject prediction model to omit a subject-specific calibration phase, the use of gel-based EEG still needs some time and effort to prepare, thereby making it impractical to use an EEG-based learning environment on a wide basis. With the recent development of dry EEG electrodes it was shown that dry electrodes provide lower signal-to-noise ratio than gel-based electrodes, but signal quality is still good enough for brain-computer interface control (Spüler, 2017). While usability of such a system could be improved by dry EEG electrodes, the increased cost can likely not be justified when using EEG-based learning environments on a broad population (e.g., in a class room). However, this technique could prove helpful for special cases in which the user suffers from learning disability or other problems. In the presented study, one subject of the experimental group suffered from test anxiety and reported to feel very comfortable using the EEG-based learning environment, as the system turned down the difficulty every time the subject was closed to feeling overwhelmed, thereby providing a good learning experience.

### 4.3. Improving the EEG-based learning environment

As this work should merely serve as a proof-of-concept to show the feasibility of an EEG-based learning environment, it should be discussed how such a system could be potentially improved.

One possible improvement could be made by not only detecting workload, but also other cognitive properties like vigilance, attention or engagement. As we already mentioned in Spüler et al. (2016), arithmetic exercises with a very high difficulty show similar EEG patterns as exercises with very low difficulty, which is likely due to a disengagement effect (Chanel et al., 2008) where subjects do not even try to solve the task. Therefore, taking additional parameters into account when adapting the learning material, seems to be advisable. Besides workload, also vigilance and engagement are important factors for solving a task correctly and learn efficiently. Decreasing vigilance is often specified as the decline in attention-requiring performance over an extended period of time. Furthermore, vigilance increases steeper in the context of difficult, compared to easy tasks. Tiwari et al. (2009) describe the interrelationship of vigilance and workload. An increasing workload is accompanied by a vigilance decrease. Engagement and workload increased as a function of task difficulty during learning and memory tasks (Berka et al., 2007). The results from previous studies (Oken et al., 2006; Berka et al., 2007; Tiwari et al., 2009) showed, the detection of various vigilance and engagement states is possible. Being able to detect these cognitive states based on the EEG could thereby improve the workload prediction and also add another layer to the learning environment where not only the learning material is presented, but also other cues could be integrated to motivate the learner when signs of disengagement or boredom are detected. Roy et al. (2013) have also shown that there is an interaction between fatigue and workload, which potentially decreases classification performance over time. As Roy et al. (2016b) have also shown that ERPs are a more robust indicator of workload than power spectral features, ERPs could be used as an additional feature to predict workload in future EEG-based learning environments. But, as ERP-based workload detection is stimulus-dependent, this approach is less flexible than using the power spectrum, which can be used for workload estimation independent of any stimuli.

## 5. Conclusion

In this paper, we presented an EEG-based learning environment, which unobtrusively detects the user's workload online and adapts the learning material accordingly to support each learner optimally. In a first study we collected EEG of subjects solving arithmetic exercises in the decimal number system. Based on this data a cross-subject prediction model was created that allows to predict the workload of other subjects without the need for a subject-specific calibration phase. This prediction model was used online to predict the workload of subjects learning arithmetic in the octal number system and the learning material was adapted promptly to keep the learner's workload in an optimal level. Utilizing the EEG-based learning environment showed a learning success similar to using a state-of-the-art system, which suggests the feasibility of an EEG-based learning environment.

## Author contributions

CW and MS conceived and designed the studies. CW programmed the online learning environment. CW collected the data. CW and MS analyzed the data. CW and MS wrote the article, partly based on the doctoral dissertation by CW, and aided by MB, WR, and PG.

### Conflict of interest statement

The authors declare that the research was conducted in the absence of any commercial or financial relationships that could be construed as a potential conflict of interest.
